# Effects of halolactones with strong feeding-deterrent activity on the growth and development of larvae of the lesser mealworm, *Alphitobius diaperinus* (Coleoptera: Tenebrionidae)

**DOI:** 10.1007/s13355-016-0411-x

**Published:** 2016-05-02

**Authors:** Maryla Szczepanik, Anna Gliszczyńska, Maksymilian Hnatejko, Beata Zawitowska

**Affiliations:** Faculty of Biology and Environment Protection, Nicolaus Copernicus University, Lwowska 1, 87-100 Toruń, Poland; Department of Chemistry, Wroclaw University of Environmental and Life Sciences, Norwida 25, 50-375 Wrocław, Poland

**Keywords:** Damascone, Antifeedants, Halolactones, Biopesticides, *Alphitobius diaperinus*

## Abstract

The effects of dietary applied of β-damascone and its synthetic derivatives γ- and δ-halolactones, which show strong antifeedant activity, on the growth and development of larvae of the lesser mealworm, *Alphitobius diaperinus* Panzer (Coleoptera: Tenebrionidae), were studied. Bioassays were performed in a dose-dependent manner. In the bioassays, oat flakes treated with 1.0, 0.5, and 0.1 % (w/v) acetone solutions of the tested compound or acetone alone as control were served as food. The experiments were conducted using 2-week-old larvae with an average body weight of 4.35–4.88 mg. High correlations between antifeedant activity and larvicidal and growth-inhibitory effects were observed. Larvae reared on diets containing the compounds (at a concentration of 1 %) with high deterrent activity were characterized by a prolonged period of development, lower body weight gain, and strong tendency for cannibalism as a result of starvation. The control larvae ended their development after 24 days with a mean body weight of 22.9 mg. At the same developmental time, the mean body weights of larvae treated with the δ-halolactones γ-chloro- and γ-bromo-δ-lactone were only 60.3 % and 43.2 % of that of the control larvae. The larval periods for larvae on the diets containing γ-chloro- and γ-bromo-δ-lactone were 33 and 41 days, respectively. The larval developmental time and body weight gain were not significantly influenced by lower doses of the compounds, with the exception of γ-bromo-δ-lactone. This compound, when applied at a concentration of 0.5 %, significantly prolonged larval development as compared to the control larvae, and caused high mortality of larvae and pupae. The adult emergence percentage was 37.51 % when this treatment was applied, as compared to 90.0 % in the control. Thus, β-damascone derivatives with a lactone ring exhibit both dose-dependent behavioral effects and post-ingestion toxicity against *A. diaperinus* larvae, and may have the potential to control this pest.

## Introduction

Numerous problems associated with the use of chemical insecticides have led researchers to look for natural agents that can be directed against insect pests. Bioactive compounds from plants are considered an ecologically safe source of environmentally friendly insecticides, and they help to reduce synthetic insecticide usage (Miresmailli and Isman 2014). These compounds with various chemical structures are accumulated in plant tissues and act as a defense system against phytopathogens and herbivores due to their antifungal, antiviral, antibacterial, and insecticidal properties (Bassolé and Juliani 2010; Ćosić et al. 2010; Pattnaik et al. 1997). Some of the defensive compounds may affect insect growth or physiology, and many are toxic, but their primary function may be to modify behavior (Bernays and Graham 1988; Rodriguez-Saona and Trumble 1999). Many of them are characterized by repellent, antioviposition, or antifeedant activities against both agricultural pests and insects that are important from a medical or veterinary perspective (Baskar et al. 2009; Koul et al. 2008; Regnault-Roger et al. 2012). Secondary plant metabolites that exhibit a high level of antifeedant activity play a more important role in insect food selection than feeding attractants or stimulants (Nawrot and Harmatha 2012). Numerous studies of natural or synthetic antifeedants show a correlation between chemical structure and antifeedant activity. The characteristic feature of antifeedants is their high specificity; their deterrent effect depends on the insect species considered (Schoonhoven 1982).

However, compounds of natural origin—while possessing many useful characteristics—can only be used to a limited extent, mainly because of their presence at only low levels in plants and the expense involved in obtaining these active ingredients. Knowledge of the structures of these compounds may facilitate the development of synthetic analogs or derivatives of them. The chemical transformation of natural compounds by incorporating the elements responsible for antifeedant activity into their structures can provide antifeedants that show rapid biodegradation and low toxicity in mammals. Components of essential oils, especially terpenes, can be used as precursors for active feeding deterrents (Dancewicz et al. 2008; Lochyński et al. 2002; Popławski et al. 2000; Szczepanik et al. 2014).

β-Damascone, also known as a rose ketone, was isolated from Bulgarian rose oil (Demole et al. 1970). It is mainly used in the perfume industry due to its pleasant smell, but it is also known for its insecticidal activity. According to Kaufman et al. (2011), β-damascone could be used against insecticide-resistant strains of the housefly, *Musca domestica* L. (Diptera: Muscidae), and other biting flies of medical and veterinary importance. Derivatives of it with a lactone ring that were synthesized in our lab showed high antifeedant activity against the lesser mealworm, *Alphitobius diaperinus* Panzer (Gliszczyńska et al. 2014). Out of the 11 compounds evaluated, three of them were particularly active against larvae of this pest: γ- and δ-chlorolactones as well as a δ-bromolactone. As described in the literature, antifeedant activity is also often associated with other biological effects against insects, such as larval growth inhibition, chronic toxicity, antioviposition, and reduced fertility (Akhtar and Isman 2004; Jeyasankar et al. 2010, 2014; Pavela et al. 2008). Studying nutritional indices such as relative consumption and growth rates after the ingestion of potential antifeedants can help to determine whether a chemical compound exhibits not only antifeedant activity but also toxicity following ingestion (Zapata et al. 2009).

The work described in the present paper is a continuation of our previous studies, and focuses on the effects of a few selected halolactones with strong deterrent activities that were obtained from β-damascone on the development of *A. diaperinus*. Our aim was to investigate if the antifeedant effects of these halolactones are long-lived and influence the larval growth, development, pupation rate, pupal and adult masses, adult emergence rate, and survival of the lesser mealworm. This insect is one of the most important and widespread pests in commercial poultry production around the world. It is capable of transmitting a large number of poultry diseases and parasites. All stages of the pest can act either as reservoirs or external carriers of serious poultry diseases, including Newcastle and Marek’s diseases, and can also act as intermediate hosts for tapeworms and protozoans (Lambkin 2001). The beetle is also known to be a pest of animal feeds, especially those kept in neglected storage rooms. This pest is usually controlled with chemical insecticides such as insect growth regulators and pyrethroids. Continuous usage of these insecticides leads to the selection of the resistant pest population (Chernaki-Leffer et al. 2011). Thus, many studies have been performed to identify natural substances or derivatives of them that can be used as insecticides with low environmental risk (Koul et al. 2008; Regnault-Roger et al. 2012). In this work, the insecticidal activities of derivatives with completely new structures were compared with the activity of the starting compound β-damascone.

## Materials and methods

### Chemicals

**Fig. 1:**
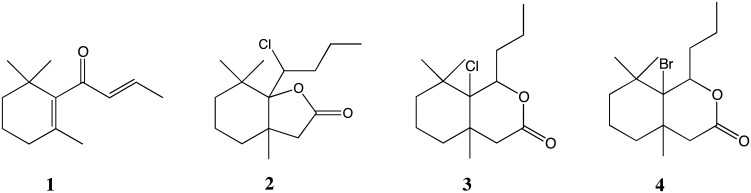
Chemical structures of the compounds (**1**–**4**) studied in this work

The structures of the compounds applied in the bioassays reported here are illustrated in Fig. 1. In this group is a natural ketone, β-damascone (**1**), as well as three halolactones synthesized from it: 7*a*-(1-chlorobutyl)-3*a*,7,7-trimethylhexahydrobenzofuran-2-one (**2**); 7*a*-chloro-3*a*,7,7-trimethyl-8-propyloctahydroizochromen-2-one (**3**), and 7*a*-bromo-3*a*,7,7-trimethyl-8-propyloctahydroisochromen-3-one (**4**) (Gliszczyńska et al. 2014). β-Damascone (**1**) was purchased from Sigma–Aldrich. The first step in the synthesis was the reduction of the double bond in the side chain of β-damascone (**1**) with LiAlH_4_ to a ketone, leading to dihydro-β-damascone, which was subsequently transformed into the corresponding allylic alcohol: dihydro-β-damascol. The Claisen–Johnson rearrangement (orthoacetate modification) of the alcohol was the key step in the synthesis. A γ,δ-unsaturated ethyl ester was the product of this rearrangement, ethyl 2-(2-butylidene-1,3,3-trimethylcyclohexyl) acetate, which was then hydrolyzed (KOH, EtOH) to 2-(2-butylidene-1,3,3-trimethyl-cyclohexyl)acetic acid. This was converted into a δ-halo-γ-lactone, 7*a*-(1-chlorobutyl)-3*a*,7,7-trimethylhexahydrobenzofuran-2-one (**2**) (18 % yield), and two γ-halo-δ-lactones, 7*a*-chloro-3*a*,7,7-trimethyl-8-propyloctahydroizochromen-2-one (**3**) (17 % yield) and 7*a*-bromo-3*a*,7,7-trimethyl-8-propyl-octahydroisochromen-3-one (**4**) (27 % yield), in bromo- and chlorolactonization processes performed under basic conditions (NBS/NCS, THF). The purities of compounds **1**–**4** were >99 % according to GC analysis.

## Bioassays 

### Insect culture

Larvae of the lesser mealworm from a laboratory colony were used in these studies. The insects were originally collected from poultry house litter in a commercial chicken farm near Toruń (53°01′N, 18°37′E, Poland). In the laboratory, the insects were kept in glass containers on a basic diet consisting of 1 part oat flakes, 1 part wheat bran, 0.01 parts brewers’ yeast, and apple halves to maintain moisture levels at ca. 60 ± 5 % r.h. The colony was kept in a rearing chamber at +29 °C in the dark.

### Feeding-deterrent activity

The feeding-deterrent activities of the compounds studied here against larvae and adults of *A. diaperinus* were determined using the standard method of choice and no-choice tests, as described previously (Gliszczyńska et al. 2014). The relative deterrence coefficient *R* was calculated using the following formula:

$$R = \frac{(C - E)}{(C + E)} \times 100,$$

where *C* and *E* are the weights of the control and treated foods consumed by the insects in the choice test, respectively. The absolute deterrence coefficient *A* was calculated using the same formula, while *C* and *E* were obtained from the no-choice test.

### Chronic larval growth, development, and survival bioassays

The effects of β-damascone and its derivatives on larval growth and development were studied by incorporating them as acetone solutions into the diet. The studies described here were performed in a dose-dependent manner. 1.0, 0.5, and 0.1 % (w/v) acetone solutions of the compounds were applied in bioassays. One gram of oat flakes was treated with 1 mL of the tested solution or acetone alone (the control) using a micropipette. This corresponded to a dose of 10.0, 5.0, or 1.0 mg of each compound per gram of diet, respectively. After solvent evaporation, oat flakes were placed in plastic containers with a capacity of 100 mL together with the moist cotton ball to keep the relative humidity below 55 %. Ten two-week-old larvae with an average body weight of 4.35–4.88 mg were placed in each container, fed ad libitum, and kept in an incubator under the same conditions as used to maintain the colony. During the developmental period, larval body weight gain and survivability were recorded at 6-day intervals. The surviving larvae received the treated food until the pupal stage. When the larva had completed their development, the resulting pupae were transferred to small plastic containers with a piece of wet cotton wool. The larval developmental times were recorded and the pupae (approximately 24 h old) were weighed. Upon successful adult emergence, before they began to feed, the body weights of the insects were also recorded. The pupation and adult emergence percentages were calculated from the formula: % pupation or adult emergence = 100 × (total number of pupae or adults formed)/(total number of larvae released) (Singh et al. 2010). The mortality of the pupae was also recorded. Each experiment was replicated three times, with 10 larvae per replicate.

### Statistical analysis

The data were statistically analyzed by means of one-way analysis of variance (ANOVA) followed by Tukey’s test (*α* = 0.05) using the Paleontological Statistics (PAST) software (Hammer et al. 2001).

## Results and discussion

The compounds tested in these studies differed in their feeding-deterrent activities. The antifeedant properties of the compounds were assessed on the basis of their deterrence coefficients. Generally, the higher the value of this index, the lower the rate of feeding (Table 1). The starting substrate, β-damascone, was the weakest antifeedant of the compounds tested. In the no-choice test, the consumption of food treated with 1 and 0.5 % concentrations of this compound was decreased by about 40 and 11 %, respectively, as compared with the control food. Chemical modification of the β-damascone structure leads to increased antifeedant activity of the resulting derivatives. During experiments, we observed structure- and dose-dependent relationships. The halolactones with a δ-lactone moiety (**3** and **4**) were the most active compounds. These differences in activity among the compounds were particularly visible in the no-choice tests where feeding was strongly suppressed. The consumption of food treated with the maximum dose of δ-halolactones was more than 7 (**3**) or 6 (**4**) times lower than the consumption of food treated with β-damascone (**1**). The larval feeding rate was reduced threefold by δ-chloro-γ-lactone (**2**) in comparison to β-damascone, while the activity of δ-bromo-γ-lactone was found to be similar to that of β-damascone, so this compound was not considered in the present studies (Gliszczyńska et al. 2014). Cutting the dosage of halolactones by half led to a decrease in deterrent activity. When 0.5 % solutions of the δ-halolactones were used, consumption increased more than sixfold (when **4** was used) and about fivefold (when **3** was used) in relation to the consumption observed with 1 % solutions. The lowest dose (0.1 %) of each compound was almost completely ineffective as an antifeedant (Table 1).

**Table 1:** Feeding-deterrent activities of the compounds studied in the choice and no-choice tests against *A. diaperinus* larvae

Dose (%)	Compound^a^	Deterrence coefficients (mean ± SE)		Consumption (%)^b^
		*R*	*A*	
1.0^c^	**1**	55.59 ± 3.41abc	30.70 ± 15.94ab	59.26 ± 17.31bcd
	**2**	97.77 ± 0.53d	67.04 ± 4.36bc	21.44 ± 8.26ab
	**3**	98.29 ± 2.96d	85.71 ± 5.30c	7.88 ± 2.64a
	**4**	80.19 ± 1.17c	82.45 ± 11.50c	9.69 ± 1.67a
0.5^c^	**1**	30.09 ± 1.66a	12.75 ± 0.18a	88.81 ± 0.44d
	**2**	84.22 ± 6.03c	29.99 ± 6.83ab	58.28 ± 9.02bcd
	**3**	65.62 ± 11.96bc	48.25 ± 9.42abc	36.59 ± 9.71abc
	**4**	84.61 ± 4.97c	29.78 ± 14.52ab	59.25 ± 15.40bcd
0.1	**3**	32.71 ± 13.05ab	11.21 ± 4.97a	80.87 ± 7.71c
	**4**	58.32 ± 15.23bc	8.18 ± 1.52a	84.98 ± 1.52d
	*F*	12.35	8.89	10.73
	*df*	9	9	9
	*p*	<0.001	<0.001	<0.001

*R* relative coefficient (choice test), *A* absolute coefficient (no-choice test)

Means followed by the same letters within each column are not significantly different (one-way ANOVA and Tukey’s test, *p* < 0.05)

^a^For structures of the compounds labeled by number in this column, see Fig. 1

^b^Data are expressed as percentages of the consumption of the control in the no-choice test

^c^From Gliszczyńska et al. (2014)

The antifeedant efficiencies of a significant number of chemical compounds depend on the dose and exposure time. After a long exposure period, some of these compounds lose their antifeedant properties due to the development of resistance in the insects that consume the contaminated food, degradation of the compounds, or habituation (Pavela 2011; Rodriguez-Saona and Trumble 1999). None of these phenomena were observed for the most potent antifeedants (i.e., δ-halolactones at a concentration of 1 %) studied in this work; the compounds showed permanent antifeedant activity across the whole test period. The antifeedant effect was rather short-lived after the application of δ-chloro-γ-lactone (**2**). While the deterrence coefficients determined in three-day assays (Gliszczyńska et al. 2014) indicated that δ-chloro-γ-lactone possesses high activity, larval feeding rates started to increase when it was used over a long period. Likely, persin presented high deterrence against larvae of *Spodoptera exigua* Hübner (Lepidoptera: Noctuidae) during the first 72 h, but after that the deterrence declined (Rodriguez-Saona et al. 1997). Therefore, studies of feeding deterrents should have a broader scope than just the determination of the deterrence coefficients.

One of the consequences of feeding inhibition is a low body-weight gain. The larval body weights of larvae treated with the maximum doses of compounds **3** and **4** were considerably lower than those of the control larvae. After 12 days of culture, the body weights of the larvae treated with δ-halolactones had increased approximately 1.5-fold, while the increase in body weight for the control larvae was about 4.5 times. Larval growth in the presence of β-damascone was only slightly slower (3.6-fold increase) than that of the control.

In the control trials, larvae ended their development after 24 days with a mean body weight of 22.9 mg. At the same time, the mean body weights of larvae treated with δ-halolactones were only 43.2 % (**4**) and 60.3 % (**3**) of the mean body weight of the control larvae (Table 2). The smallest body weight gain of the larvae was observed after the application of γ-bromo-δ-lactone (**4**). Admittedly, this compound showed slightly weaker antifeedant activity in the no-choice test than γ-chloro-δ-lactone (**3**) did, but this difference was not significant (Table 1). Thus, these compounds showed the strongest inhibitory activities towards both feeding and larval growth when administered at their maximum doses. However, their ability to reduce the pest population through their antifeedant action is crucial.

**Table 2:** Growth of *A. diaperinus* larvae reared on a diet treated with different concentrations of β-damascone and halolactones

Dose (%)	Compound^a^	Body weights (mg) ± SE^b^ on particular post-treatment days					
		0	6	12	18	24	BWRC^c^
	Control	4.44 ± 0.09a	10.60 ± 0.78ab	19.82 ± 0.22a	22.41 ± 1.18a	22.9 ± 0.00a	–
1.0	**1**	4.88 ± 0.28a	10.43 ± 0.90ab	15.93 ± 1.47bcd	20.85 ± 1.78abc	21.80 ± 0.00ab	95.20
	**2**	4.41 ± 0.02a	8.85 ± 0.31b	13.54 ± 0.09cd	16.52 ± 0.67cd	19.75 ± 1.39ab	86.20
	**3**	4.53 ± 0.14a	4.62 ± 0.17c	7.03 ± 0.30e	9.85 ± 1.08e	13.81 ± 1.70c	60.30
	**4**	4.35 ± 0.10a	4.47 ± 0.57d	6.11 ± 0.70e	8.40 ± 1.48e	9.90 ± 2.29d	43.20
0.5	**1**	4.41 ± 0.05a	9.16 ± 0.18b	17.21 ± 0.95bc	20.65 ± 1.80abc	21.28 ± 2.06ab	92.90
	**2**	4.54 ± 0.05a	11.19 ± 0.53a	17.56 ± 0.72ab	21.40 ± 0.31ab	22.21 ± 1.05a	96.99
	**3**	4.44 ± 0.07a	8.40 ± 0.37c	13.05 ± 0.71d	16.65 ± 0.85bcd	17.00 ± 0.69bc	74.24
	**4**	4.38 ± 0.03a	5.49 ± 0.14c	9.09 ± 0.79e	12.46 ± 0.81de	14.66 ± 0.45c	64.03
0.1	**3**	4.37 ± 0.11a	9.45 ± 0.66ab	16.98 ± 0.53abc	19.09 ± 0.77abc	21.36 ± 0.59ab	93.27
	**4**	4.59 ± 0.09a	8.83 ± 0.82b	15.97 ± 0.57bcd	18.94 ± 1.06abc	19.83 ± 0.68ab	86.58
	*F*	–	24.95	37.84	23.65	16.37	
	*df*	–	10	10	10	10	
	*p*	–	<0.001	<0.001	<0.001	<0.001	

Means followed by the same letters within a column are not significantly different (one-way ANOVA and Tukey’s test, *p* < 0.05)

^a^For structures of the compounds labeled by number in this column, see Fig. 1

^b^Each value is the mean of three replicates, each set up with ten larvae (*n* = 30)

^c^Body weight relative to control (%)

In our studies, larval growth was also affected by the application of a lower dose (0.5 %) of δ-halolactones **3** and **4**, despite a decrease in antifeedant activity in the no-choice test. In the choice test, the activities of these compounds remained high as a result of their effects on the chemoreceptors. This is typical of many antifeedants. Usually, the mean values of the relative coefficients are higher than those of the absolute coefficients. The conclusions drawn from the choice test must be confirmed by comparing the results obtained in the choice test with those obtained in the no-choice test. In this test, larvae only had access to the treated food, so only strong stimulation of their chemoreceptors would stop them feeding. The no-choice situation is often more representative of an agricultural system (Koul 2004; Nawrot and Harmatha 2012). Our previous studies showed that larvae of the lesser mealworm are more sensitive to antifeedants in the choice test than in the no-choice situation (Szczepanik et al. 2008, 2014). Only in the case of a very strong feeding deterrent are both deterrence coefficients very high, and just such a situation was observed after applying the δ-halolactones **3** and **4** at a concentration of 1 % (Table 1).

On the other hand, a concentration of 0.5 % δ-chloro-γ-lactone (**2**) had no effect on the growth of larvae. The deterrence coefficients of lactones **2** and **4** were almost identical in both tests, but these lactones had different impacts on larval growth. This may suggest that the antifeedant activity of compound **2** is relatively short-acting, and/or that compounds **3** and **4** have toxic effects following ingestion. A smaller reduction in the growth rate was observed after the application of lactone **3**, which showed higher deterrent activity in the no-choice test. The differences in these parameters from those of lactone **4** were not significant, but they were significant in comparison with those of the control. The growth of larvae that consumed δ-halolactones at a concentration of 0.1 % was similar to that of the control (Table 2).

Strong antifeedant activities of natural compounds or their derivatives along with a reduction in larval growth are relatively commonly observations. Limonoids, phragmalin, and mexicanolide obtained from *Khaya senegalensis* (Desr.) A. Juss (Meliaceae) exhibited strong antifeedant activity against the cotton leafworm, *Spodoptera littoralis* Boisduval (Lepidoptera: Noctuidae), and inhibited larval growth (El-Aswad et al. 2003). Extract of the seeds of *Melia volkensii* Gürke (Meliaceae) appears to act as a strong antifeedant and an effective growth inhibitor of cabbage looper, *Trichoplusia ni* Hübner (Lepidoptera: Noctuidae), and the armyworm, *Pseudaletia unipuncta* Haworth (Lepidoptera: Noctuidae) (Akhtar and Isman 2004). Likewise, two avocadofurans with antifeedant activity against the generalist insect herbivore *S. exigua* were found to reduce the larval growth of late-stadium larvae, and significantly extended their development times, even at sublethal concentrations (Rodriguez-Saona and Trumble 1999). Chronic inhibition of larval growth after the application of feeding deterrents may be due to not only an antifeedant effect but also a behavioral deterrence combined with a post-ingestive toxic effect (Isman et al. 1989; Pavela et al. 2008; Stefanazi et al. 2011; Zapata et al. 2009). When some sesquiterpene lactones were incorporated into the diet of *Tenebrio molitor* L. (Coleoptera: Tenebrionidae), its conversion of ingested food in insect biomass decreased (Rosiński et al. 1988).

The compounds studied here do not appear to be toxic because we did not observe any dead larvae shortly after applying the compounds to the food. The strong reduction in the pupation rate after applying δ-halolactones **3** and **4** at 1 % concentration was due to a high level of cannibalism among the larvae, triggered by the effect of starvation. When there were high deterrence coefficients, it was difficult to determine whether there was a post-ingestive effect because the larvae barely fed. When they are not willing to accept the treated food made available to them, the starving larvae sought out other sources of food. This phenomenon is often observed among insects reared under unfavorable conditions (lack of food, water, high density, etc.) (Richardson et al. 2010). After the application of antifeedants, the more sensitive and weaker larvae were eaten by the stronger ones before pupation. This alternative food contributed to the growth and survival of a few larvae and their formation of pupae. *A. diaperinus* is an omnivorous insect; it can eat both plant and animal foods. The level of cannibalism observed was dose-dependent. In the control trials, this phenomenon was observed sporadically, as it was after applying the lower doses of the compounds, especially a concentration of 0.1 %. In trials where the concentration of the δ-halolactone was 0.5 %, the rate of cannibalism was <15 % (10–13.3 %). According to Bouayad et al. (2013), cannibalistic behavior was observed in insects exposed to plant extracts with antifeedant activity. For example, extracts of *Centaurium erythraea* Rafn (Gentianaceae) and *Rosmarinus officinalis* L. (Lamiaceae) induced marked cannibalism (20–25 %) between larvae of *Plodia interpunctella* Hübner (Lepidoptera: Pyralidae), similar to that observed in starved larvae (30 %). Furthermore, Rharrabe et al. (2007) reported that the presence of harmaline, a β-carboline alkaloid that is a secondary metabolic compound in plants, in the diet of larvae of *P. interpunctella* caused the appearance of cannibalistic behavior.

Larvae with low body weights were characterized by a significantly prolonged period of development in comparison with the control ones (Table 3). The duration of the larval period was correlated with the antifeedant activity of the tested compound—the starvation induced by the compound resulted in growth inhibition. The highest dose of δ-halolactone, which was correlated with strong antifeedant activity, led to the greatest prolongation of larval development. The larval periods for larvae fed on diets containing γ-chloro- and γ-bromo-δ-lactone were 33 and 41 days, respectively, while that for the control group was only 21 days (Table 3). Prolonged development caused by starvation was also seen in *T. ni*, for which there was a direct correlation between larval duration and the level of starvation (Tignor and Eaton 1986). Larval developmental time was not significantly influenced by compound **2**, which exhibited relatively low antifeedant activity. After treatment with a moderate dose (0.5 %), larval duration was only increased significantly by γ-bromo-δ-lactone (**4**); treatment with the other compounds at a moderate dose led to similar larval durations to that seen for the control (Table 3).

**Table 3:** Effects of different concentrations of β-damascone and halolactones on the development of *A. diaperinus* larvae

Dose (%)	Compound^a^	Larval duration (days ± SE)	Pupation (% ± SE)	Pupal weight (mg ± SE)	Adult emergence (% ± SE)	Adult weight (mg ± SE)
	Control	21.0 ± 1.73a	96.7 ± 3.33a	19.17 ± 1.06a	90.0 ± 5.77a	14.94 ± 0.55a
1.0	**1**	25.0 ± 2.0ab	70.0 ± 5.77bc	18.16 ± 0.74ab	40.0 ± 10b	15.10 ± 1.05a
	**2**	24.0 ± 1.15ab	60.0 ± 8.82c	16.71 ± 0.91ab	40.0 ± 5.77b	14.18 ± 0.84a
	**3**	33.0 ± 3.46c	30.0 ± 5.77d	15.06 ± 0.85ab	26.7 ± 3.33b	11.14 ± 0.55a
	**4**	41.0 ± 2.65c	20.0 ± 5.77d	14.15 ± 1.08b	16.7 ± 3.33b	11.86 ± 1.41a
0.5	**1**	23.50 ± 0.65ab	92.5 ± 2.5ab	17.56 ± 0.71ab	85.51 ± 5.00a	14.61 ± 0.72a
	**2**	20.33 ± 0.58a	93.33 ± 3.3ab	16.31 ± 1.27ab	90.0 ± 5.77a	14.60 ± 0.51a
	**3**	25.51 ± 0.96ab	77.51 ± 2.5abc	15.41 ± 0.66ab	70.0 ± 8.16a	13.58 ± 0.51a
	**4**	30.25 ± 2.36bc	75.0 ± 7.5c	14.51 ± 0.57b	37.51 ± 9.46b	12.05 ± 0.87a
0.1	**3**	21.75 ± 0.48a	87.54 ± 4.79ab	17.63 ± 0.58ab	82.5 ± 3.23a	14.69 ± 0.83a
	**4**	22.25 ± 1.49a	95.0 ± 2.89a	16.39 ± 0.60ab	77.53 ± 7.51a	13.45 ± 0.32a
	*F*	18.62	30.49	2.61	18.2	2.49
	*df*	10	10	10	10	10
	*p*	<0.001	<0.001	0.018	<0.001	0.014

Values are means of three replicates, each set up with ten larvae (*n* = 30)

Means followed by the same letters within each column are not significantly different (one-way ANOVA and Tukey’s test, *p* < 0.05)

^a^For structures of the compounds labeled by number in this column, see Fig. 1

Pupal and adult weights were influenced by treatment with any of the tested compounds, but the pupal weight was reduced to the greatest degree by the δ-lactones, especially γ-bromo-δ-lactone (**4**). This compound was effective at both high and moderate doses (1.0 and 0.5 %). The weights of the pupae that received either of these treatments were the lowest. Poor antifeedants had a relatively weak reductive effect on the body weights of pupae and adults as well as pupation percentages in comparison with those of the control (Table 3). Thus, the quantity and quality of the available food are key influences on the growth, development, and reproduction of insects (Liu et al. 2013). Cereal flours, especially rice flour, reduced the growth of larvae, pupae, and adults of *A. diaperinus*, lowered pupation and adult emergence, and significantly lengthened the developmental time (Hosen et al. 2004).

In our studies, adult emergence rates varied and were lower than the pupation rates, even in the controls, but the number of adults that emerged was strongly affected in three treatments: those with β-damascone and lactone **2** at 1 % concentration and that with lactone **4** at 0.5 % concentration. Some of the adults that emerged from the β-damascone-treated larvae were deformed and died soon after emergence. The difference between pupation rate and adult emergence rate was 30.0 % in this treatment, while it was 6.7 % in the controls. This is not surprising, because monoterpenoids very often act as insect growth regulators. Doses of certain essential oils or their components that are too low to kill insects can still induce genotoxic effects and trigger various types of malformations (Halder et al. 2012; Karr and Coats 1988). For example, *Argemone mexicana* L. (Papaveraceae) extracts reduced adult emergence and increased pupal mortality in *S. litura* (Malarvannan et al. 2008). Crude extract of *Aristolochia elegans* Mast. (Aristolochiaceae) produced pronounced deformations and malformations in the larval, pupal, and adult stages of *S. litura* (Arivoli and Tennyson 2013). A high pupal mortality (35.5 %) was also recorded when lactone **4** was applied at 0.5 % concentration or lactone **2** (20 %) was used at a 1 % dose, although they did not cause any malformations. The cause of pupal mortality could be post-ingestion toxicity. The effects of this toxicity could build up over time. After applying the compounds, larvae and pupae died slowly several days after treatment. A post-ingestive toxic effect of polygodial on *S. littoralis* larvae, which did not induce early death, was reported by Zapata et al. (2009).

β-Damascone was recently discovered to be a potential insecticide against several mosquito species and the housefly (Kaufman et al. 2011). However, neither β-damascone nor its derivatives showed acute toxicity towards *A. diaperinus* larvae when they were incorporated into the diet at 1 % concentration. Maybe the toxicity of this compound depends on the insect species considered or the method of application. Differences in susceptibility to insecticides/chemicals among insect species depend primarily upon the diverse biological mechanisms involved in the metabolism of toxic substances. Different species may metabolize or excrete toxic compounds in different ways and at different rates. Sensitivity to β-damascone even varies among different species of mosquitoes (Kaufman et al. 2011). The main reason for a lack of gastric/dietary toxicity of the β-damascone derivatives when applied at the maximum dose to food could be their antifeedant activity, which could lead to relatively low doses of the compound being ingested by the larvae. More dead larvae and pupae were observed when the antifeedant activity of the treatment was lower.

In conclusion, our results indicate that 1 % solutions of δ-halolactones with the damascone skeleton are strong antifeedants, and therefore show the potential to be used as an agent for controlling *A. diaperinus*. These compounds are also very good antifeedants against *A. diaperinus* adults, even at lower doses: 0.5 and 0.1 % (Gliszczyńska et al. 2014). These lower doses are less effective against larvae, with the exception of γ-bromo-δ-lactone (**4**), which significantly prolonged larval development as compared to the control larvae when applied at 0.5 % concentration, and its application resulted in both larval and pupal mortality. The adult emergence percentage in this treatment was 37.51 %, as compared to 90.0 % in the control larvae. Thus, β-damascone derivatives with a lactone ring present dose-dependent behavioral effects and post-ingestive toxicities against *A. diaperinus*. Moreover, lactone derivatives of β-damascone were also found to be very active regulators of the behavior of peach-potato aphids, *Myzus persicae* (Sulz) (Gabryś et al. 2015). They may represent a new group of insecticides that readily biodegrade in the natural environment. The microorganism-catalyzed decomposition of halolactones is based on two main mechanisms: hydrolytic dehalogenation, leading to the substitution of the halogen by a hydroxyl group (Janssen 2004); or elimination of the halogen atom and the formation of a double bond (Grotowska and Wawrzeńczyk 2002; Janssen et al. 2001).
